# Does Positive Thinking Help during Difficult Pandemic Times? The Role of Positive Orientation in the Relationship between Fear of COVID-19 and Perceived Stress

**DOI:** 10.3390/ejihpe13010011

**Published:** 2023-01-11

**Authors:** Joanna Dymecka, Rafał Gerymski, Anna Machnik-Czerwik, Aleksandra M. Rogowska

**Affiliations:** Institute of Psychology, University of Opole, 45-040 Opole, Poland

**Keywords:** fear of COVID-19, perceived stress, positive orientation, global pandemic, mediation analysis

## Abstract

The COVID-19 pandemic has become a huge challenge for the modern world. How people perceive themselves and their coping abilities is important for their mental health and well-being. One of the traits that may be important in effectively coping with difficulties is positive orientation: a stable cognitive disposition that is the opposite of depression and is associated with a positive perception of oneself, one’s life, events, and the future. This study aimed to verify the role of positive orientation in the relationship between fear of COVID-19 and perceived stress. A sample of 907 Polish people took part in this study. FOC-6, P Scale, and PSS-10 questionnaires were used in the presented cross-sectional study. The analysis showed that women scored higher in fear of COVID-19 and perceived stress scores than men (Cohen’s *d* indicated a moderate effect). There was no significant difference in the levels of positive orientation. P Scale results were significantly related to fear of COVID-19 (small effect) only for the whole studied sample, and not for women and men considered separately. Fear of COVID-19 was positively related to the perceived stress score (moderate effect). Positive orientation was negatively related to the PSS-10 scores (also moderate effect) for all tested groups. Fear of COVID-19 and positive orientation were significant predictors of perceived stress scores. The mediation effect of positive orientation was statistically significant, but the effect size was marginally small. Positive orientation is an important predictor of perceived stress, which could be related to the COVID-19 pandemic. People with a positive orientation better cope with the challenges of the pandemic and are optimistic about the future. Working on positive orientation can improve well-being and reduce tension, which is extremely important in difficult pandemic times.

## 1. Introduction

The COVID-19 pandemic has become a huge challenge for the modern world. Since its announcement, over 600 million people have been infected, and 6.5 million have died [1]. During the COVID-19 pandemic, people were afraid of infection, complications, and own death. They were also afraid that someone close to them would get sick or die. People were afraid of contact with others who could transmit the virus. The fear was also aroused by quarantine, the need for isolation, hospitalization, and the lack of access to healthcare facilities [2,3]. In addition to the risk of being infected, the pandemic has caused many social, family, and work limitations. As a result, it has become a significant cause of stress and mental health problems [4]. Stress during a pandemic is probably associated with both the fear of getting infected and its consequences, as well as the feeling of helplessness, frustration, loneliness, many changes in professional and social functioning, and the financial crisis [5,6].

Many studies focused on the strengths of the individuals and their personal resources, which allowed them to effectively deal with the challenges posed by the pandemic. How people perceive themselves and their coping abilities is important for their mental health and well-being [7,8,9]. One of the traits that may be important in effective coping with difficulties is a positive orientation. This basic personality trait reflects a general tendency to perceive life experiences with a positive attitude [10,11]. According to the theory of positivity (POS), positive orientation is a stable global cognitive disposition to optimistic thinking, a positive assessment of life satisfaction and self-esteem, affecting people’s feelings, cognition, and actions [10,11,12]. POS decreases the risk of depression and is associated with a positive perception of oneself, one’s life, events, and the future [12]. Capara et al. [10,11,12] showed that a positive cognitive triad promotes well-being and flourishing, allowing for effective coping with negative life events, diseases, or traumatic situations, and may protect against negative psychological consequences of life events [13,14,15,16]. People with high POS compensate for negative affect by fostering positive affectivity. They tend to evaluate external reality positively and overestimate their performance and attributes, coping with difficulties better than those with low POS [14].

Previous research demonstrated that people experiencing depression and anxiety reported low levels of subjective well-being, and the relationship between mental health and well-being is reciprocal [17]. Recent research showed a negative relationship of life satisfaction (one of the POS components) with fear of COVID-19 (or anxiety) and perceived stress [7,8,18,19,20]. Fear of COVID-19 is also positively related to stress [2,4,6,21,22]. Seligman [23] showed evidence that optimism modulates perceived stress, as well as depression, emotion, and mood, and can protect from physical illness (including cardiovascular diseases, hypertension, high cholesterol) and unhealthy lifestyle consequences (obesity, smoking, and excessive alcohol use). Similarly, the review study by Scheier and Carver [24] demonstrated that optimism is negatively correlated with various physical health outcomes, including mortality, heart failure, and stroke. From the biological point of view, optimists have better immune systems. They tend to cope with stress better than pessimists, generating less stress hormone (cortisol), fibrinogen, and high heart rate variability [23,25]. The study evidenced that a positive future orientation is a mediator between traumatic experiences and mental health [26].

The current study will examine the associations between fear of COVID-19, perceived stress, and POS. Endler [27] proposed the multidimensional interaction model (MIM), which can explain the interaction mechanism between fear of COVID-19, POS, and perceived stress. When a person with a certain personality characteristic (e.g., anxiety trait, dispositional optimism) perceives a stressful situation (e.g., COVID-19 pandemic) as a threat or danger, this can lead to an increase in arousal (e.g., heightened fear of COVID-19), which in turn can lead to biochemical and physiological changes and the perception of stress. All associations between personal characteristics (e.g., vulnerability to fear or POS disposition), situational factors (e.g., COVID-19 pandemic risk and restrictions level), individual perception of the situation (e.g., fear or pleasure), change in arousal (increasing or decreasing), and responses to changes in arousal (e.g., perceived stress) are bidirectional and interact with each other. Due to the MIM, it is expected that personal traits (such as POS) can mediate the relationship between fear of COVID-19 and perceived stress.

Sirgy [17] distinguished several theories that can explain the mediating role of POS on mental and physical health, including stress and fear response to the challenging environment. The affective disposition theory [28] argues that affective states (e.g., positive emotions such as hope, happiness, love, optimism, or negative emotions such as fear, guilt, jealousy, anger, or sadness) influence well-being alongside situational factors. Furthermore, the instrumental and temperament theories suggested that extraversion and neuroticism predispose people to certain cognitive and behavioral reactions to various situations. For example, extrovert individuals are more prone to positive stimuli and positive affect, while neurotics are more sensitive to punishment, negative emotions, and mood. Happiness is also genetically determined. According to the top-down theory [29], happy people tend to overestimate themselves, which biases their perception of outcomes in specific domains (including perceived stress). In contrast, unhappy people are likelier to provide further weight to their worst domain (so they can experience greater stress response).

The other possible explanation of mediating role of POS in the relationship between fear of the COVID-19 pandemic and perceived stress is Lazarus’s transactional stress model [30,31]. Stress is the result of an interaction between a person and the environment. The arousal and stress response depends on specific features of the environment (more or less stable, predictable, threatening) as well as on characteristics of a person (self-efficacy, self-esteem, individual dispositions, and coping abilities). Primary appraisal refers to the subjective experience of the event as stressful in terms of irrelevant stressors, i.e., positive or dangerous (challenge or threat). Secondary appraisal refers to an assessment of a person’s ability to cope. Coping with stress results from the transaction between environmental demands and current personal or social resources (sufficient or insufficient). Fear of COVID-19 can be considered a type of primary appraisal in this study. High anxiety levels are experienced in highly anxious individuals (a biologically determined trait, related to introversion and neuroticism). This study’s secondary appraisal can be understood as a reference to stable POS that defines past and future actions in different situations. The secondary appraisal (positive *versus* negative orientation) can lead to changes in stress response (decrease or increase, respectively to POS levels). As such, it is expected that POS will serve as a mediator in the relationship between fear of COVID-19 and perceived stress.

Consistent with the appraisal theory, happy people tend to appraise their life events more positively than unhappy persons. Moreover, even if the situation is objectively “neutral”, happy individuals perceive it more positively. Therefore, they can experience lower levels of perceived stress [17]. The attribution theory of happiness [32] suggests that well-being is related to the optimistic attribution style (success is considered as a result of internal, stable factors, including personality traits, self-efficacy, and self-esteem). Conversely, people feel more unhappy when they use pessimistic attribution (internal, stable) to explain their failures. If people think they have little control over good events that happen to them, they may experience a lower level of subjective well-being than when they feel responsible for those events.

**Figure 1:**
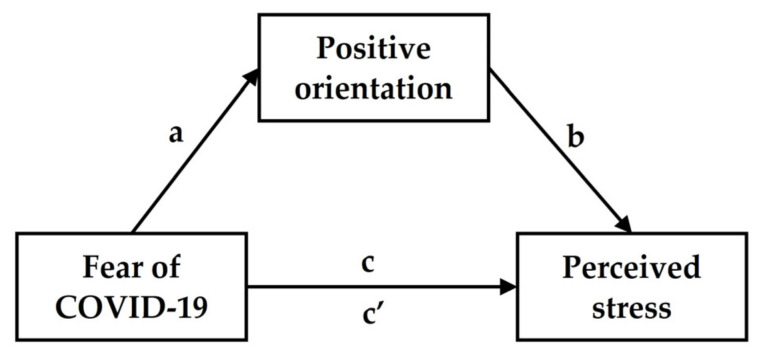
The hypothesized model of the mediating effect of positive orientation on the relationship between fear of COVID-19 and perceived stress.

Based on previous research [2,4,6,7,8,17,18,19,20,21,22,23,24,25,26], we assumed that both fear of COVID-19 and positive orientation would significantly predict the perceived stress experienced during a global pandemic. However, the mediating effect of positive orientation on the relationship between fear of COVID-19 and perceived stress will be examined in this study for the first time (Figure 1), consistent with MIM [27] and transactional model of stress [30,31], as well as the positive orientation theories [17,28,29,32].

## 2. Materials and Methods

### 2.1. Participants

Of the 907 Polish people who participated in this study, 385 men and 522 women. The mean age of the study population was 39.28 years old. Detailed sociodemographic data of the studied sample are presented in Table 1.

### 2.2. Procedure

The presented study took place between March and May 2020. Due to the global pandemic, the study participants were recruited via the internet. Elderly people who were digitally excluded could complete the survey by phone (*n* = 11). Only people above 18 years of age took part in this study. All respondents were informed about the anonymity of the study and gave their informed consent to participate in it. The presented project received the positive recommendation of the Bioethics Committee of the University of Opole (KEBN 15/2021).

### 2.3. Measures

The Fear of COVID-19 Scale (FOC-6) has been used in the presented study [2]. It consists of 6 items on a 5-point scale (1 = “definitely disagree”; 5 = “completely agree”). Higher FOC-6 scores indicate higher fear of COVID-19. In the presented study, FOC-6 obtained good reliability (Cronbach’s alpha = 0.83; McDonald’s total omega = 0.84).

The Perceived Stress Scale (PSS-10) measure has also been selected for this study [33,34]. It consists of 10 items on a 5-point scale (0 = “never” and 4 = “very often”). Higher PSS-10 scores indicate higher perceived stress. In the presented study, PSS-10 obtained good reliability (Cronbach’s alpha = 0.85; McDonald’s total omega = 0.86).

The levels of the participants’ positive orientation were measured with a P Scale [35,36]. It consists of 8 items on a 5-point scale (1 = “I strongly disagree”’ and 5 = “I strongly agree”). The higher the P Scale score, the higher the level of positive orientation. In the presented study, the P Scale obtained good reliability (Cronbach’s alpha = 0.87; McDonald’s total omega = 0.87).

### 2.4. Statistical Analysis

Several statistical analyses were performed to verify hypotheses. Descriptive statistics were performed to check the parametric properties of the data (*M*, *SD*, *Mdn*, skewness, and kurtosis). Moreover, homogeneity of variance was under control to examine the gender differences in fear of COVID-19, positive orientation, and perceived stress, using the independent samples *t*-test. The Cohen’s *d* was considered to check the effect size for this analysis. Correlation and multiple linear regression analyses were performed to examine the associations between fear of COVID-19, positive orientation, and perceived stress. Next, the mediation analysis was performed using Model 4 mediation of PROCESS 4.1 [37]. The bootstrap technique was implemented with a 5000 resampling [38] to increase the stability and accuracy of models.

## 3. Results

### 3.1. Group Homogeneity Analysis

First, it was decided to check whether the tested sample of study participants was a homogeneous group. The *t*-test analysis showed that women scored higher in fear of COVID-19 and perceived stress scores than men (Cohen’s *d* indicated a moderate effect). There was no significant difference in the levels of positive orientation. More detailed data are shown in Table 2. Based on the provided results, we have decided to calculate the correlation analysis with gender as a grouping variable.

### 3.2. Correlation, and Mediation Analysis

Pearson’s *r* correlation indicated that positive orientation was significantly related to fear of COVID-19 only for the whole studied sample, but the size of the effect was small. On the other hand, fear of COVID-19 was positively related to the perceived stress score (moderate effect). Moreover, positive orientation was negatively related to the PSS-10 scores (also moderate effect) for all tested groups. For more detailed results, see Table 3.

According to the hypothesis, we examined the mediating role of positive orientation on the relationship between fear of COVID-19 and perceived stress. Fear of COVID-19 and positive orientation were significant predictors of perceived stress scores. The mediating effect was statistically significant, but the effect size was marginally small. For more detailed results, see Table 4.

## 4. Discussion

This study analyzed the relationship between fear of COVID-19, positive orientation, and perceived stress. It is one of the first studies to analyze positive orientation in the context of the relationship between fear of COVID-19 and perceived stress. In our study, the average positive orientation score was similar to the average results of Polish standardization tests in adults [36]. This means that the respondents remained positive and optimistic despite the pandemic and probably felt satisfied with their life. There were no differences in the level of positive orientation due to the sex of the respondents. In contrast, women have been shown to experience greater fear of COVID-19 and greater stress during a pandemic than men. Previous studies have already described higher scores for women on the PSS-10 scale [39,40]. This finding may be related to gender differences in coping with stress. Women have a greater tendency to worry and a higher level of anxiety. Numerous studies [41] have shown that women report greater intensity of fear and anxiety symptoms than men. Kendler et al. [42] showed that women are more sensitive to stressful life events. A study by Dalgard et al. [43] explains that women are more prone to problems in the social network because they are characterized by a more affiliated style of functioning and greater involvement in the household and family matters. During the first waves of the pandemic, social contact was significantly reduced.

Consistent with the hypothesis and previous research [2,4,6,7,8,17,18,19,20,21,22,23,24,25,26], this study found fear of COVID-19 and positive orientation as predictors of perceived stress. Many researchers point out that stress levels during a pandemic were higher than usual [2,4,6]. At the beginning of the pandemic, people were probably afraid of COVID-19 due to the fear of infection, complications, hospitalization, quarantine, isolation, or possibly of their own or loved ones [2]. On the other hand, our study showed that positive orientation was an important predictor of stress. Still, it did not play a significant role in the relationship between fear of COVID-19 and stress. The importance of positive orientation as a significant predictor of perceived stress was also confirmed in other studies [44]. Research shows that an optimistic attitude to life and high self-esteem are important predictors of stress [45]. People who are satisfied with their lives and themselves have great faith in the future and experience less stress, despite difficulties. For those people, the tension associated with a difficult situation turns into stress less frequently. It is related to an optimistic view of reality and the awareness that many difficulties can be dealt with. Research shows that positive orientation plays an important role in selecting strategies for coping with difficult situations. People with a high positive orientation are more likely to use problem-focused coping. These strategies, stimulating positive emotions, are essential for adapting to unfavorable circumstances [46]. It is also indicated that positive orientation is relatively constant. It was shown that the level of positive orientation did not change significantly over four years. This indicates that people’s judgments about themselves, the world, and their future are relatively constant and impact an individual’s well-being and mental functioning [47,48]. People who perceived their life and future positively before the pandemic probably remained optimistic during the pandemic, which means they felt much less stressed.

The study showed that there was a statistically significant but marginally weak relationship between fear of COVID-19 and positive orientation. Only a few studies have examined the association between positive orientation and fear of COVID-19. Another Polish study found no relationship between these two variables [49]. Concerning the COVID-19 pandemic, there are considerations of both fear and anxiety. Fear is an adaptive mechanism for animals and humans, fundamental to survival.

Finally, we confirmed the mediating role of POS on the relationship between fear of COVID-19 and perceived stress. It can be explained using stress and well-being theories. Consistent with MIM [27], there is an interaction between environmental factors (such as the COVID-19 pandemic), personality traits (individual differences in fear and stress response, dispositional optimism), perception of the current situation understood as a threat or danger, and changes in arousal, leading to the perception of stress. Consistent with MIM [27], disposition to POS can play mediating role in the association between fear of COVID-19 and perceived stress. Consistent with affective disposition theory [28], people with predispositions to a high reaction of fear to various situations, including the COVID-19 pandemic, may also tend to increase the stress response and decrease POS. The tendency of negative emotions to dominate is genetically determined and related to other personality traits, such as extraversion and neuroticism [50]. Unfortunately, we did not control for personality traits in the present study. In future studies, extraversion and neuroticism could be included in the mediation model together with POS. On the other hand, happy perceive all situations in a better light, so they experience fewer levels of fear and stress, consistent with the top-down theory [29].

Taking into account the Lazarus’s transactional stress model [30,31], the mediating role of POS can be explained as a secondary appraisal in the perceived stress-environment (COVID-19 pandemic) transaction. After a fear response to COVID-19 (as a primary appraisal), a person refers to his or her resources to cope with the problem in a second step. The second appraisal includes personal disposition to POS, which is based on past experiences and predicts future behavior. Therefore, fear of COVID-19 can be considered a type of primary appraisal in this study. As appraisal theory shows, happy people appraise all life events more positively than unhappy people [17], so they experience less fear and stress. According to the attribution theory of happiness [32], those who use the optimistic attribution style experience positive emotions more frequently than negative. In contrast, those who use pessimistic attribution may see the situations in the worst light and experience high fear and stress levels.

Positive orientation is a cognitive variable underlying self-esteem, optimism, and life satisfaction. It is associated with adaptive functioning and positive self-assessment. It is also associated with well-being, happiness, and positive emotionality [35,36,48]. It can be assumed that the relationship between fear of COVID-19 and stress will also be mediated by other variables (e.g., personal resources), such as the sense of coherence, mental hardness and resilience, and self-efficacy. Resilience resources can directly be related to coping with difficult situations. On the other hand, positive orientation is more related to assessing oneself, one’s life, and the future. The weak relationship between fear of COVID-19 and a positive orientation may suggest that fear of being infected will be more related to the ability to cope rather than to a positive or negative attitude towards oneself and one’s own life. However, it is also worth emphasizing that the stress itself during the COVID-19 pandemic is associated not only with the possibility of being infected but also with significant changes in social life, isolation, economic problems, and changes in lifestyle and work. A person with a high level of positive orientation may cope better with the challenges of the pandemic because of their optimism and positive expectations about the future.

The presented study is not free of limitations. A longitudinal study would be necessary to verify whether the suggested direction of the influence is accurate. Additionally, the presented sample is a result of a convenient sampling method. Such a sample sometimes lacks the power to identify sociodemographic group differences [8]. The proposed mediation model and potential group differences should thus be confirmed by a second study using population-based sampling. Moreover, the demographic variables, such as education and place of residence, could be examined as potential confounders. Although the mediating effect was statistically significant, the effect size of the mediation analysis was very small, which decreased the theoretical and empirical contribution of this study. It is possible that the latent composite score of positive orientation is inappropriate for the mediating role. Therefore, life satisfaction, dispositional optimism, and self-esteem should be considered as separate constructs in the future. Moreover, the other variables (e.g., extraversion and neuroticism as a personality trait, self-efficacy, sense of coherence, grit, hope, or other resilience dimensions) could be considered as potential mediators in future studies.

## 5. Conclusions

Positive orientation is an important predictor of perceived stress, which could be related to the COVID-19 pandemic. People with a positive orientation better cope with the challenges of the pandemic and are optimistic about the future. However, it is not related to the level of fear of COVID-19. Positive orientation as a significant predictor of perceived stress may be included when planning therapeutic interventions for people experiencing particularly severe difficulties during and after a pandemic. Zuffianò et al. [51] and Park et al. [44] suggest that working on positive orientation can improve well-being and reduce tension, which is extremely important in difficult pandemic times.

## Figures and Tables

**Table 1:** Characteristics of the studied sample (*N* = 907).

Variable	Category	*M* (*SD*) or *n* (%)
Age	Overall	39.28 (15.30)
	Women	38.59 (15.22)
	Men	40.21 (15.38)
Gender	Women	522 (57.55)
	Men	385 (42.45)
Education	Elementary School	28 (3.09)
	Vocational	65 (7.17)
	High School	347 (38.26)
	University	467 (51.49)
Place of Residence	Village	220 (24.26)
	Town	410 (45.20)
	City	277 (30.54)
Professional Activity	Unemployed	268 (29.55)
	Employed	639 (70.45)

**Table 2:** Results of the *t*-test analysis (*N* = 907).

Variable	Women		Men		*t* (905)	*p*	95% CI		*d*
	*M*	*SD*	*M*	*SD*			LL	UL	
Fear of COVID-19	22.65	5.45	19.73	5.94	7.69	<0.001	2.179	3.673	0.51
Positive orientation	29.40	5.75	29.80	5.82	−1.04	0.301	−1.165	0.360	0.07
Perceived stress	21.89	5.71	18.77	5.60	8.22	<0.001	2.380	3.873	0.55

Note: *M* = mean, *SD* = standard deviation, CI = confidence interval, LL = lower level, UL = upper level, *d* = Cohen’s d effect size.

**Table 3:** Results of the Pearson’s *r* correlation analysis (*N* = 907).

Sample	Variable	*M*	*SD*	1.	2.
Total	1. Fear of COVID-19	21.41	5.84		
	2. Positive orientation	29.57	5.78	−0.08 *	
	3. Perceived stress	20.57	5.87	0.34 ***	−0.44 ***
Women	1. Fear of COVID-19	22.65	5.45		
	2. Positive orientation	29.40	5.75	−0.07	
	3. Perceived stress	21.89	5.71	0.31 ***	−0.45 ***
Men	1. Fear of COVID-19	19.73	5.94		
	2. Positive orientation	29.80	5.82	−0.08	
	3. Perceived stress	18.77	5.60	0.28 ***	−0.44 ***

Note: * *p* < 0.05; *** *p* < 0.001.

**Table 4:** Results of the mediation analysis for perceived stress (*N* = 907).

Mediation								
Path			Symbol	*Beta*	*SE*	*p*	LLCI	ULCI
*X*	→	*M*	a	−0.08	0.03	0.015	−0.144	−0.015
*M*	→	*Y*	b	−0.41	0.03	<0.001	−0.477	−0.365
*X* (*M*)	→	*Y*	c’	0.31	0.02	<0.001	0.254	0.365
Indirect Effect			a * b	0.03	0.01		0.005	0.061

Note. *X* = Fear of COVID-19, *M* = Positive orientation, *Y* = Perceived stress, LLCI = lower-level confidence interval, ULCI = upper-level confidence interval.

## Data Availability

The data can be made available from the corresponding author upon reasonable request.

## References

[1] World Health Organization Coronavirus Disease (COVID-19): Weekly Epidemiological Update on COVID-19-5 October 2022. https://www.who.int/publications/m/item/weekly-epidemiological-update-on-covid-19---5-october-2022.

[2] Dymecka J., Gerymski R., Machnik-Czerwik A. (2021). Fear of COVID-19 as a buffer in the relationship between perceived stress and life satisfaction in the Polish population at the beginning of the global pandemic. Health Psychol. Rep..

[3] Fardin M.A. (2020). COVID-19 and anxiety: A review of psychological impacts of infectious disease outbreaks. Arch. Clin. Infect. Dis..

[4] Dymecka J. (2021). Psychosocial effects of the COVID-19 pandemic. Neuropsychiatria Neuropsychologia Neuropsychiatry Neuropsychology.

[5] Brooks S.K., Webster R.K., Smith L.E., Woodland L., Wessely S., Greenberg N., Rubin G.J. (2020). The psychological impact of quarantine and how to reduce it: Rapid review of the evidence. Lancet.

[6] Polizzi C., Lynn S.J., Perry A. (2020). Stress and coping in the time of COVID-19: Pathways to resilience and recovery. Clin. Neuropsychiatry.

[7] Dymecka J., Gerymski R., Machnik-Czerwik A. (2022). How does stress affect life satisfaction during the COVID-19 pandemic? Moderated mediation analysis of sense coherence and fear of coronavirus. Psychol. Health Med..

[8] Dymecka J., Gerymski R., Machnik-Czerwik A., Derbis R., Bidzan M. (2021). Fear of COVID-19 and life satisfaction: The role of the health-related hardiness and sense of coherence. Front. Psychiatry.

[9] Bidzan M., Bidzan-Bluma I., Szulman-Wardal A., Stueck M., Bidzan M. (2020). Does self-efficacy and emotional control protect hospital staff from COVID-19 anxiety and PTSD symptoms? Psychological functioning of hospital staff after the announcement of COVID-19 coronavirus pandemic. Front. Psychol..

[10] Caprara G.V. (2009). Positive orientation: Turning potentials into optimal functioning. Eur. Health Psychol..

[11] Caprara G.V., Steca P., Alessandri G., Abela J.R., McWhinnie C.M. (2010). Positive orientation: Explorations on what is common to life satisfaction, self-esteem, and optimism. Epidemiol. Psych. Sci..

[12] Caprara G.V., Alessandri G., Trommsdorff G., Heikamp T., Yamaguchi S., Suzuki F. (2012). Positive orientation across three cultures. J. Cross. Cult. Psychol..

[13] Russo C., Dell’Era A., Zagrean I., Danioni F., Barni D. (2022). Activating self-transcendence values to promote prosocial behaviors among adolescents during the COVID-19 pandemic: The moderating role of positive orientation. J. Genet. Psychol..

[14] Caprara G.V., Eisenberg N., Alessandri G. (2017). Positivity: The dispositional basis of happiness. J. Happiness Stud..

[15] Caprara G.V., Alessandri G., Caprara M. (2018). The associations of positive orientation with health and psychosocial adaptation: A review of findings and perspectives. Asian J. Soc. Psychol..

[16] Ferreira M.J., Sofia R., Carreno D.F., Eisenbeck N., Jongenelen I., Cruz J.F.A. (2021). Dealing with the pandemic of COVID-19 in Portugal: On the important role of positivity, experiential avoidance, and coping strategies. Front. Psychol..

[17] Sirgy M.J. (2012). The Psychology of Quality of Life: Hedonic Well-Being, Life Satisfaction, and Eudaimonia.

[18] Rogowska A.M., Ochnik D., Kuśnierz C., Chilicka K., Jakubiak M., Paradowska M., Głazowska L., Bojarski D., Fijołek J., Podolak M. (2021). Changes in mental health during three waves of the COVID-19 pandemic: A repeated cross-sectional study among Polish university students. BMC Psychiatry.

[19] Rogowska A.M., Kuśnierz C., Ochnik D. (2021). Changes in stress, coping styles, and life satisfaction between the first and second waves of the COVID-19 pandemic: A longitudinal cross-lagged study in a sample of university students. J. Clin. Med..

[20] Rogowska A.M., Kuśnierz C., Bokszczanin A. (2020). Examining anxiety, life satisfaction, general health, stress and coping styles during COVID-19 pandemic in Polish sample of university students. Psychol. Res. Behav. Manag..

[21] Ochnik D., Rogowska A.M., Arzenšek A., Benatov J. (2022). Longitudinal predictors of coronavirus-related PTSD among young adults from Poland, Germany, Slovenia, and Israel. Int. J. Environ. Res. Public Health.

[22] Rogowska A.M., Ochnik D., Kuśnierz C. (2022). Revisiting the multidimensional interaction model of stress, anxiety and coping during the COVID-19 pandemic: A longitudinal study. BMC Psychol..

[23] Seligman M.E.P. (2011). Flourish: A Visionary New Understanding of Happiness and Well-Being.

[24] Scheier M.F., Carver C.S. (2018). Dispositional optimism and physical health: A long look back, a quick look forward. Am. Psychol..

[25] Seligman M.E.P. (2000). Optimism, pessimism, and mortality. Mayo Clin. Proceed..

[26] Zhang J., Zhao G., Li X., Hong Y., Fang X., Barnett D., Lin X., Zhao J., Zhang L. (2009). Positive future orientation as a mediator between traumatic events and mental health among children affected by HIV/AIDS in rural China. AIDS Care.

[27] Endler N.S. (1997). Stress, anxiety and coping: The multidimensional interaction model. Can. Psychol..

[28] Diener E., Smith H., Fujita F. (1995). The personality structure of affect. J. Pers. Soc. Psychol..

[29] Diener E. (1984). Subjective well-being. Psychol. Bull..

[30] Lazarus R.S., Arnold W.J. (1968). Emotions and adaptation: Conceptual and empirical relations. Nebraska Symposium on Motivation.

[31] Lazarus R.S., Folkman S. (1984). Stress, Appraisal, and Coping.

[32] Schwarz N., Clore G.L. (1983). Mood, misattribution, and judgments of well-being: Informative and directive functions of affective states. J. Pers. Soc. Psychol..

[33] Cohen S., Kamarck T., Mermelstein R. (1983). A global measure of perceived stress. J. Health Soc. Behav..

[34] Juczyński Z., Ogińska–Bulik N. (2009). Narzędzia Pomiaru Stresu i Radzenia Sobie ze Stresem [Tools for Measuring Stress and Coping with Stress].

[35] Caprara G.V., Alessandri G., Eisenberg N., Kupfer A., Steca P., Caprara M.G., Abela J. (2012). The positivity scale. Psychol. Assess..

[36] Łaguna M., Oleś P., Filipiuk D. (2011). Orientacja pozytywna i jej pomiar: Polska adaptacja skali orientacji pozytywnej [positive orientation and its measurement: Polish adaptation of positive orientation scale]. Stud. Psychol..

[37] Hayes A.F. (2017). Introduction to Mediation, Moderation, and Conditional Process Analysis: A Regression-Based Approach.

[38] Preacher K.J., Hayes A.F. (2008). Asymptotic and resampling strategies for assessing and comparing indirect effects in multiple mediator models. Behav. Res. Methods.

[39] Cohen S., Janicki-Deverts D. (2012). Who’s stressed? Distributions of psychological stress in the United States in probability samples from 1983, 2006, and 2009. J. Appl. Soc. Psychol..

[40] Remor E. (2006). Psychometric properties of a European Spanish version of the perceived stress scale (PSS). Span. J. Psychol..

[41] Uhlenhuth E.H., Paykel E.S. (1973). Symptom intensity and life events. Arch. Gen. Psychiatry.

[42] Kendler K.S., Thornton L.M., Prescott C.A. (2001). Gender differences in the rates of exposure to stressful life events and sensitivity to their depressogenic effects. Am. J. Psychiatry.

[43] Dalgard O.S., Dowrick C., Lehtinnen V., Vazquez-Barquero J.L., Wilkinson C.P., Ayuso-Mateos J.L., Page H., Dunn G. (2006). Negative life events, social support, and gender differences in depression. A multinational community survey with data from the ODIN study. Soc. Psychiatry Psychiatr. Epidemiol..

[44] Park M.S.A., Goto N., Kennedy A., Raj S., Dutson A., Park L., Sovet L. (2021). Positive orientation, job satisfaction and psychological well-being of mental health practitioners in Malaysia. Psychol. Health Med..

[45] Saleh D., Camart N., Romo L. (2017). Predictors of stress in college students. Front. Psychol..

[46] Kupcewicz E., Jóźwik M. (2019). Positive orientation and strategies for coping with stress as predictors of professional burnout among Polish nurses. Int. J. Environ. Res. Public Health.

[47] Alessandri G., Caprara G.V., Tisak J. (2012). A unified latent curve, latent state-trait analysis of the developmental trajectories and correlates of positive orientation. Multivar. Behav. Res..

[48] Alessandri G., Caprara G.V., Tisak J. (2012). The unique contribution of positive orientation to optimal functioning: Farther explorations. Eur. Psychol..

[49] Chodkiewicz J., Gola M. (2021). Fear of COVID-19 and death anxiety: Polish adaptations of scales. Adv. Psychiatry Neurol..

[50] Lucas R.E., Diener E., Diener E. (2009). Personality and subjective well-being. The Science of Well-Being: The Collected Works of Ed Diener.

[51] Zuffianò A., López-Pérez B., Cirimele F., Kvapilová J., Caprara G.V. (2019). The positivity scale: Concurrent and factorial validity across late childhood and early adolescence. Front. Psychol..

